# Morphometric analysis of the patterns of calcaneal facets for the talus in Serbian population

**DOI:** 10.1371/journal.pone.0240818

**Published:** 2020-10-29

**Authors:** Nikola Vučinić, Gordana Teofilovski-Parapid, Mirela Erić, R. Shane Tubbs, Dragana Radošević, Bojana Jovančević

**Affiliations:** 1 Department of Anatomy, Faculty of Medicine, University of Novi Sad, Novi Sad, Serbia; 2 Faculty of Medicine, University of Belgrade, Belgrade, Serbia; 3 Department of Neurosurgery, Tulane Center for Clinical Neurosciences, Tulane University School of Medicine, New Orleans, LA, United States of America; 4 Department of Anatomical Sciences, St. George’s University, St. George’s, Grenada; 5 Department of Structural & Cellular Biology, Tulane University School of Medicine, New Orleans, LA, United States of America; 6 Faculty of Medicine, University of Novi Sad, Novi Sad, Serbia; PLOS ONE, UNITED KINGDOM

## Abstract

Literature describes different patterns of calcaneal facets for the talus in terms of whether some calcaneal facets are connected or separated from each other or completely absent. The aim of this study was to establish the patterns of calcaneal facets for the talus, to calculate their total area, and to analyse the data with respect to gender. The study involved 59 calcanei which were photographed. The patterns of calcaneal facets noted in this study were compared with the patterns from the literature. ImageJ program was used to measure different parameters on calcanei. The pattern 1 was the most commonly found in the study sample (45.76%), then the pattern 2 (40.68%), and finally the pattern 3 (13.56%). That order of frequencies is the same in both sexes. The patterns 1 and 2 have a larger contact surface for the talus in comparison to the pattern 3. Male bones have a larger contact surface for the talus than female bones. The sum of the pattern 1 and pattern 3 frequencies was high. Knowing the frequency of different patterns of calcaneal facets for the talus in a certain population is important for orthopaedic surgeons when performing foot osteotomy.

## Introduction

The calcaneus is the largest bone of the skeleton of the foot. There are three calcaneal facets for the talus on the upper side of the calcaneus: the anterior, middle, and posterior facet [1]. The three calcaneal facets for the talus and the three talar facets for the calcaneus participate in the construction of the talocalcaneonavicular joint and subtalar joint, and the stability of these joints depends on the facet characteristics [2]. The back side of the calcaneus corresponds to the tuber calcanei, through which most of the body weight is transferred to the surface [1]. Anterior, middle, and posterior calcaneal facets for the talus often exhibit variability in their presence and position. Recent studies performed in different populations [3–5] describe a few patterns of calcaneal facets for the talus in relation to whether one calcaneal facet is connected to another calcaneal facet, separated, or completely missing. Some studies have shown that individuals with certain morphological variations of calcaneal facets for the talus have a greater predisposition to the development of subtalar arthritis [3, 4]. In addition to that, in the biological identification of a skeleton, the determination of sex is considered the most important step [6]. The bones of pelvis and skull are most often examined for this purpose [7]. However, if these bones are damaged or missing, sex can be determined based on the morphological characteristics of the calcaneus [8–10], but it is still a matter of debate whether the key differences between sexes include the presence, position, or average facets area of the calcaneus. Steele (1976) calculated the average values of the parameters for determining the gender, and we use them in our study as relevant [11].

The aims of the study were to determine the frequency of the patterns of calcaneal facets for the talus in Serbian population, to calculate their total articular area, and finally, to analyze the obtained data in relation to gender in order to potentially establish certain differences.

## Materials and methods

The study was performed in accordance with the Declaration of Helsinki as the statement of ethical principles for medical research. The research was approved by the Ethics Committee of the Faculty of Medicine, the University of Novi Sad, Serbia, (the approval number: 01-39/269/1; the date of approval: 29.12.2017), which is the institution that handles cadavers for research and educational purposes. The persons were informed about future researches prior to their body donation, and they accepted examinations in the form of a written consent. None of the authors had access to patient data identifications. The study was conducted at the Faculty of Medicine in Novi Sad and involved 59 dry human calcanei with well-preserved calcaneal facets for the talus. The calcanei belonged to persons who resided in the Republic of Serbia, the Province of Vojvodina. The calcanei were photographed by a digital camera (Canon EOS-1D X Mark II).

Gender is established on the basis of the average values of five parameters that are commonly used for the purpose of determining calcanei:

1. Maximum length (maxl) is the distance between the most prominent point on the articular surface for the cuboid bone (anteriorly) and the most prominent point on the tuber calcanei (posteriorly) [11].
2. Minimum width (minw) is the shortest width projected through the body of the calcaneus. It is most often measured in front of the tuber calcanei and back in relation to the posterior facet for the talus [11].
3. Body height of calcaneus (bh) is measured from the lowest point on the tuber calcanei to the highest point on the posterior facet for the talus [11].
4. Load arm length (lal) is the distance between the most prominent point on the articular surface for the cuboid bone (anteriorly) and the most prominent point on the posterior facet for the talus (posteriorly) [11].
5. Value lal/maxl: This parameter is determined by the quotient of the load arm length and the maximum length [8].

The pattern of calcaneal facets for the talus was established by comparison with the patterns from the literature [3, 5]. Different parameters for determining the sex and size of calcaneal facets for the talus were measured using the ImageJ program (National Institute of Health, USA, http://rsbweb.nih.gov/ij). All the photographs and measurements were performed by the same person.

All collected data were subsequently statistically processed using Statistical Package for Social Sciences–SPSS 21, and the results are shown graphically. Student's t test was used to determine the difference between groups. Statistically significant difference was considered if p<0.05.

## Results

Of the total sample, 41 calcanei belonged to males (69.49%) and 18 to females (30.51%). There were 34 right (57.63%) and 25 left bones (42.37%).

**Fig 1:**
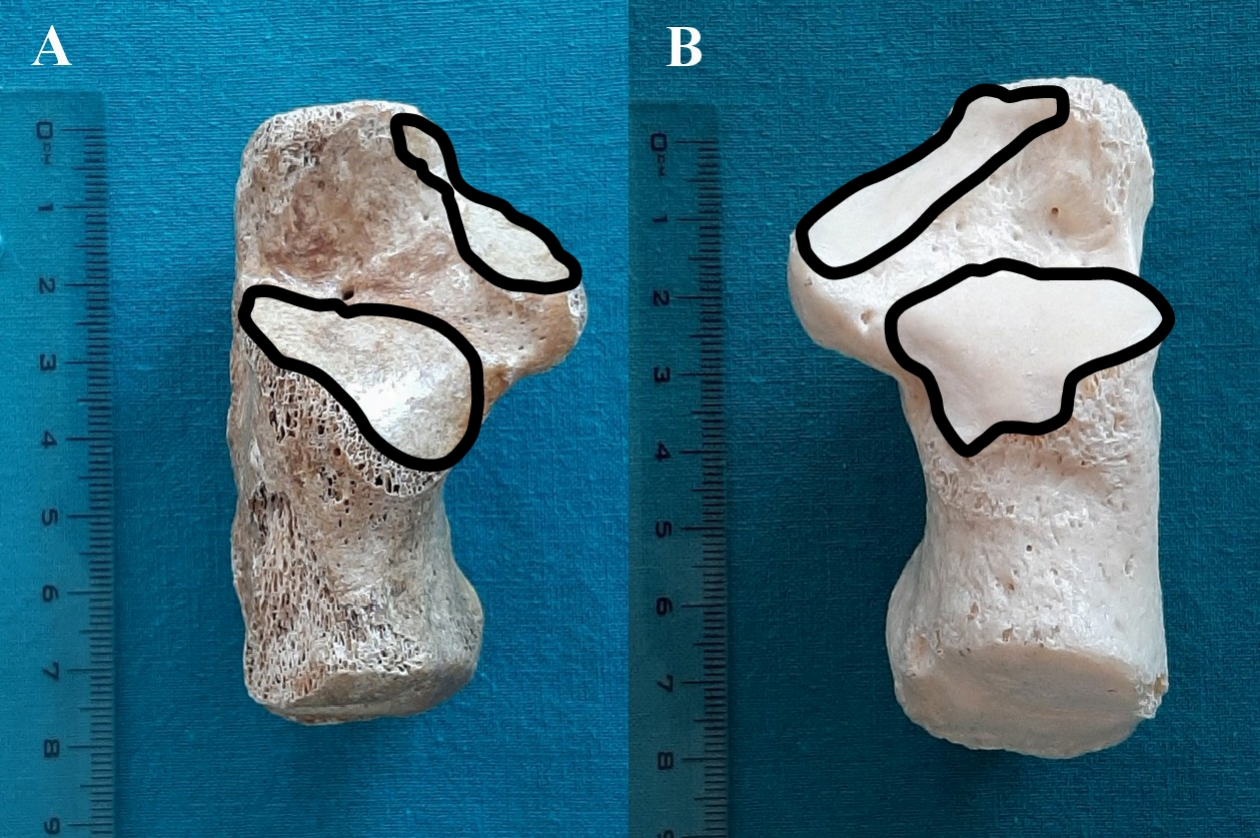
Pattern 1 of calcaneal facets for the talus: (A) pattern 1a, (B) pattern 1b.

We have described several patterns of calcaneal facets for the talus:

The pattern 1: Anterior and middle facets are connected, while the posterior facet is separated. This pattern was the most common in our sample and was observed in 27 bones. From this number, the pattern 1a (Fig 1A), in which the anterior and middle facets were connected with constriction, was present in 11 bones; the pattern 1b (Fig 1B), in which the anterior and middle facets were connected without constriction, was established in 16 bones.

**Fig 2:**
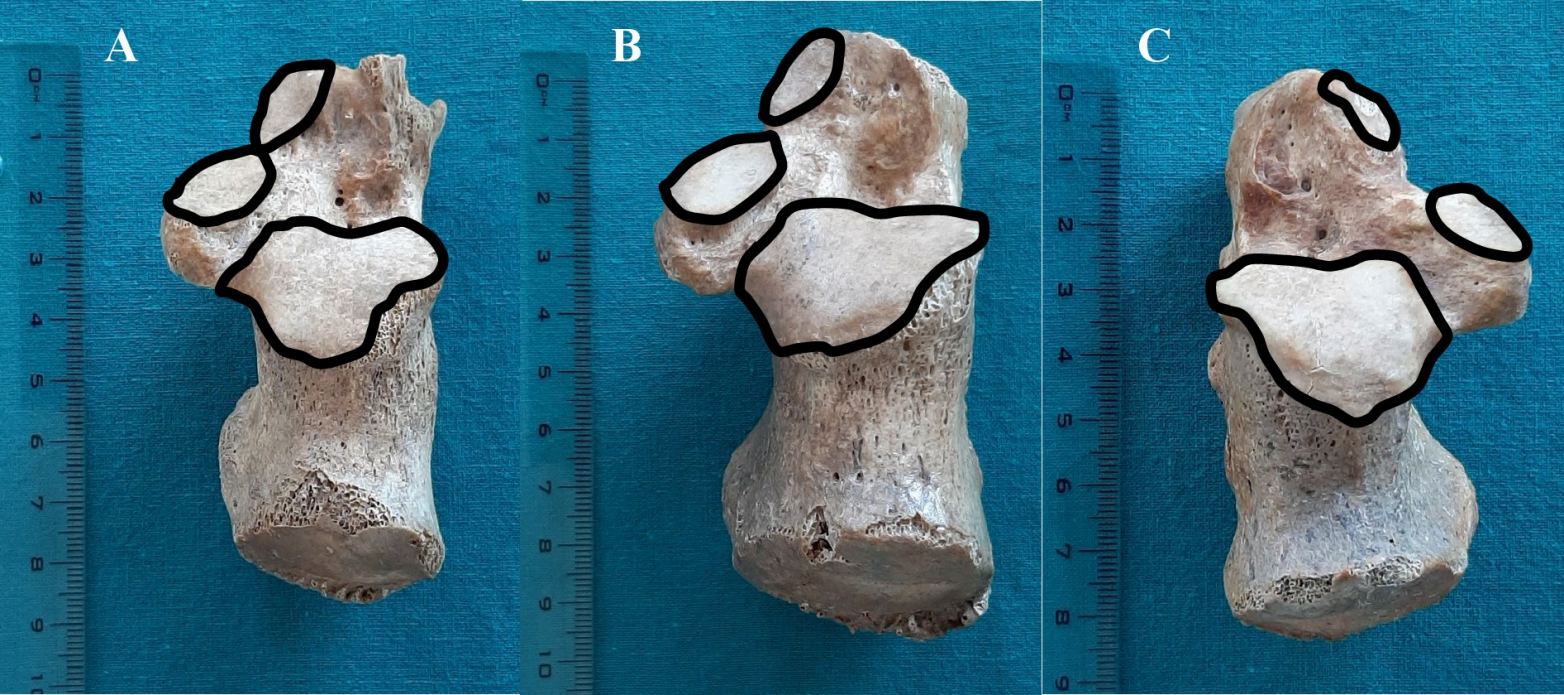
Pattern 2 of calcaneal facets for the talus: (A) pattern 2a, (B) pattern 2b, (C) pattern 2c.

The pattern 2: All three facets are clearly separated. The pattern 2 was present in 24 bones. From this number–the pattern 2a (Fig 2A), where the distance between the anterior and the middle facets was less than 2 mm, was noted in 7 bones; the pattern 2b (Fig 2B), in which the mentioned distance was between 2 mm and 5 mm, was observed in 10 bones; the pattern 2c (Fig 2C), where the distance was greater than 5 mm, was also seen in 7 bones.

**Fig 3:**
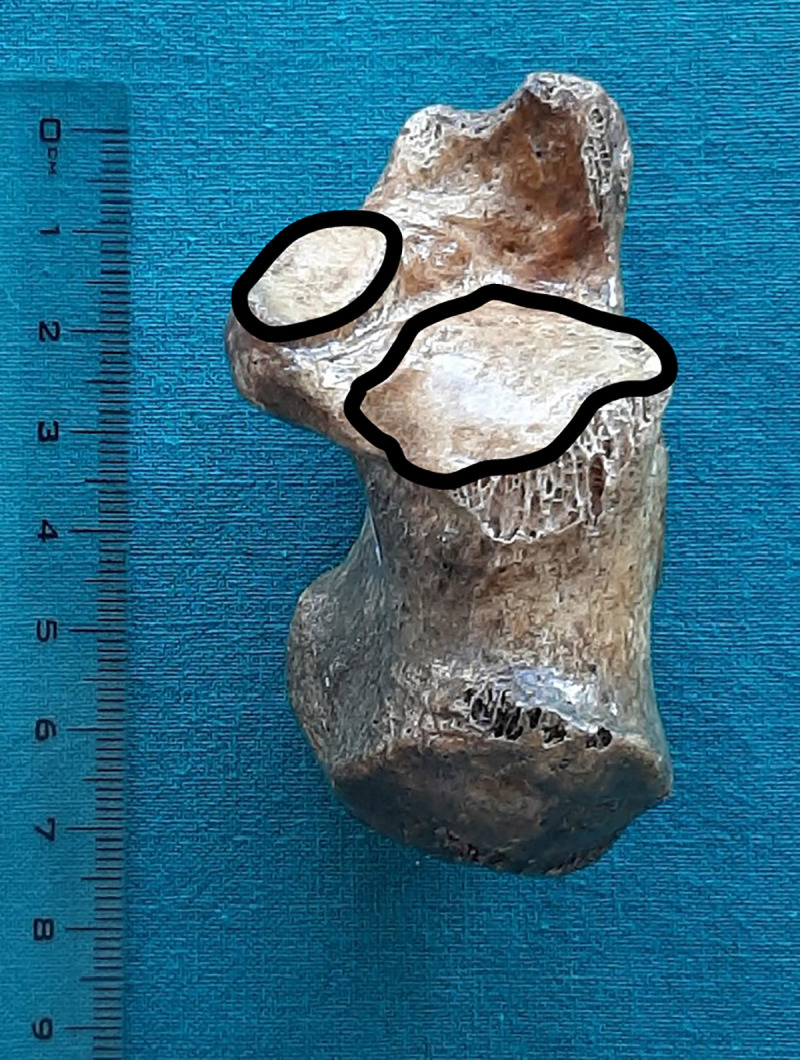
Pattern 3 of calcaneal facets for the talus.

The pattern 3: There was no anterior facet, while the middle and posterior facets were separated. The pattern 3 (Fig 3) was observed in 8 bones.

**Fig 4:**
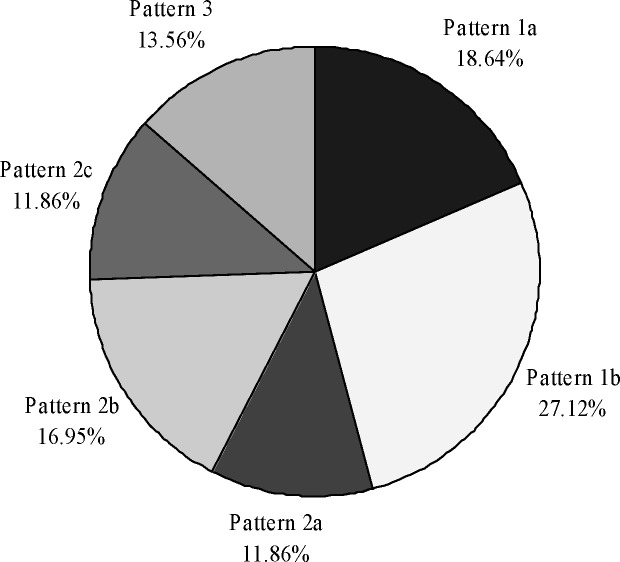
The frequency of different patterns of calcaneal facets for the talus in our sample.

The frequency of patterns of calcaneal facets for the talus is shown in Fig 4.

In the male bones, the pattern 1 was represented with 18 bones (43.90%)–of which the pattern 1a was in 7 bones and the pattern 1b in 11 bones, then the pattern 2 with 17 bones (41.46%)–wherein the pattern 2a was in 4 bones, the pattern 2b in 7 bones, and the pattern 2c in 6 bones, while the pattern 3 was present in 6 bones (14.63%). In the female bones, the most common pattern, the pattern 1, was present in 9 bones (50.00%)–of which the pattern 1a was in 4 bones and the pattern 1b in 5 bones, then the pattern 2 in 7 bones (38.89%)–wherein the pattern 2a was in 3 bones, the pattern 2b in 3 bones, and the pattern 2c in 1 bone, and the pattern 3 in 2 bones (11.11%).

Among the right bones, the most common was the pattern 2, present in 15 bones (44.12%). The pattern 1 was observed in 14 bones (41.17%). The pattern 3 was the rarest, found in 5 bones (14.71%). Among the left bones, the pattern 1 was represented with 13 bones (52.00%), followed by the pattern 2 with 9 bones (36.00%), while the pattern 3 was present in 3 bones (12.00%).

The average values of the area of calcaneal facets for the talus were 862 mm^2^ for the pattern 1, 856 mm^2^ for the pattern 2, and 726 mm^2^ for the pattern 3. By comparing the total area of the present calcaneal facets for the talus, we found that there was no statistically significant difference between the pattern 1 and the pattern 2 (t = 1.68, p>0.05). A statistically significant difference in the total area of the present calcaneal facets was established between the pattern 1 and the pattern 3 (t = 1.78, p<0.05), as well as between the pattern 2 and the pattern 3 (t = 1.77, p<0.05).

Analyzing the average values of the area of the calcaneal facets for the talus, it has been found that the male calcanei had a significantly larger total articular surface for the talus (917 mm^2^) than the female calcanei (668 mm^2^). The difference in the area of calcaneal facets between the genders was statistically significant (t = 1.67, p<0.05).

## Discussion

The dominant pattern of calcaneal facets for the talus in our population is the pattern 1, while the second most common is the pattern 2. This sequence of frequency of the most common patterns of calcaneal facets for the talus is also present in the populations of India (the pattern 1–67%, the pattern 2–26%) [12], Egypt (the pattern 1–63%, the pattern 2–30%) [13], United States (the pattern 1–54%, the pattern 2–27%) [14] and Pakistan (the pattern 1–63%, the pattern 2–28%) [15].

The results of our study do not show similarity to the data obtained for two western European countries where similar studies were conducted, and where there is a characteristic pattern 2 dominance of the calcaneal facets for the talus. In a study of British authors [16], the pattern 2 was present in 67% of the examined calcanei, while the pattern 1 was present in 33% of the bones. A study in Belgium shows that the pattern 2 was present in 64% of calcanei and the pattern 1 in 25% of bones [17]. However, it is possible that studies in the other populations of eastern Europe, close to this research, would show similarity in the dominant pattern of the calcaneal facets for the talus.

The rarely present pattern 3 occurs in our population with 8 calcanei (13.56%), while in the studies in Britain [16] and Pakistan [15] this pattern does not appear at all.

In the sample of our study, we did not have calcanei with the pattern 4 of calcaneal facets for the talus, in which all three calcaneal facets are connected in one, and the pattern 5, in which the middle calcaneal facet is connected to the posterior facet, while the anterior calcaneal facet is present and separated [18]. These two patterns occur extremely rarely in other populations [12], or they are not present at all [3, 4]. The exception is a study in Pakistan where the pattern 4 frequency is as much as 9% [15].

Analyzing the gender differences in the pattern of calcaneal facets for the talus in our sample, it can be noticed that males have the patterns 1 and 2 in the very approximate percentages, while in females, there is the dominancy of the pattern 1. In both sexes, the patterns 1 and 2 are significantly more present than the pattern 3. For that very reason, there were no significant differences in the position of calcaneal facets for the talus between sexes (the most common was the pattern 1, then the pattern 2, and finally the pattern 3). A more comprehensive comparison of men and women in terms of possible differences in the frequency of calcaneal facets patterns could be performed if we had a higher number of female bones, but, in our study, there were only 18 such bones.

By comparing the average values of the area of the calcaneal facets for the talus between the patterns 1, 2, and 3 present in the sample, it is noted that the pattern 1 and the pattern 2 have a very similar overall area, while the pattern 3 has a smaller area than the other two patterns. The reason for this difference is probably in the absence of the anterior calcaneal facet in the pattern 3.

When considering the differences in the overall average area of the calcaneal facets for the talus between the genders, it can be seen that the males have a larger area of articular facets than the females for approximately one third, which was expected to be determined, since the parameters for determining the sex on the male calcanei were higher than the same parameters in females. Searching the literature, we did not find a study that examines gender differences in relation to the patterns and average area of calcaneal facets for the talus.

In the literature, we have found that people with the pattern 2 of calcaneal facets for the talus have a lower predisposition for the development of subtalar arthritis than those with other patterns of calcaneal facets for the talus [3]. In the pattern 2, the three separated calcaneal facets form an "osseous tripod" for the talus, especially for the head of the bone, and, in this way, more effectively prevent movements that can lead to trauma and subsequent development of osteoarthritis [19]. This signifies that people with other patterns of calcaneal facets for the talus are more susceptible to develop subtalar arthritis (such as the pattern 1 and the pattern 3 in our sample). On the other hand, the total area of calcaneal facets for the talus is another important factor that affects the stability of the joint, and the pattern of calcaneal facets for the talus with the smallest overall articular area is, at the same time, the most unstable [5].

It is appropriate to mention the accessory anterolateral talar facet which articulates with the calcaneus and which also has clinical implications. Aydıngöz et al. [20] in their MRI study found this variation was present in 32.7% of patients with ankle pain and in 26% of volunteers without it. Accessory anterolateral talar facet may contribute to painful talocalcaneal impingement in persons with pes planus [20].

Knowing the frequency of different patterns of calcaneal facets for the talus in a certain population is particularly important for orthopaedic surgeons when performing osteotomy and the bone graft interposition in the correction of pes planus [4, 21]. In this procedure, it is important to determine the distance between the anterior and middle calcaneal facets for the purpose of positioning the retractor into the right position, because the osteotomy line usually goes through an equal distance between these facets [22]. This technique is suitable for patients with the pattern 2 and the pattern 3 of calcaneal facets for the talus, because of separated or absent facets. Contrary to that, when surgeons treat a patient with the pattern 1 (with connected anterior and middle facets), a suitable modification of osteotomy may be required [3, 23]. When subtalar implants or protheses are considered as a treatment option for a patient, 3D modeling with the use of imaging (generally in the form of computed tomography) is usually employed and it gives personalized characteristics for the best modeling or selection of an implant.

The limitation of this study relates to a smaller sample of bones, in particular females. The next study could be extended by increasing the number of bones. In addition, we did not have the data on the identity of the bones, and, therefore, we could not interpret any differences in the pattern of calcaneal facets between the two calcanei of the same person. Because anatomic construction of the calcaneal articular facets influences the biomechanics of the foot, further investigations into this topic are needed in order to improve the knowledge about calcaneal articular facets variations in different populations.

## Conclusions

The pattern 1 was the most common in the study sample, followed by the pattern 2, and the pattern 3. That order of frequencies is the same in both sexes. The sum of the pattern 1 and the pattern 3 frequencies (59.32%) in this study was high. In addition to that, the patterns 1 and 2 have a larger contact surface for the talus compared to the pattern 3. Additionally, male bones have a larger contact surface for the talus than female bones. Knowing the frequency of different patterns of calcaneal facets for the talus in a certain population is particularly important for orthopaedic surgeons when performing osteotomy.
